# Autonomous Lightning Strike Detection and Counting System Using Rogowski Coil Current Measurement

**DOI:** 10.3390/s25082563

**Published:** 2025-04-18

**Authors:** Arthur F. Andrade, Giovanny M. B. Galdino, Ronimack T. Souza, Newton S. S. M. Fonseca, Antonio F. Leite Neto, Edson G. Costa, Eden L. Carvalho Junior

**Affiliations:** 1 Faculty of Engineering, Languages and Social Sciences at Seridó, Federal University of Rio Grande do Norte (UFRN), Currais Novos 59380000, RN, Brazil; 2 Electrical Engineering Department, Federal University of Campina Grande (UFCG), Campina Grande 58429900, PB, Brazil; giovanny.galdino@ee.ufcg.edu.br (G.M.B.G.); ronimack@dee.ufcg.edu.br (R.T.S.); newton.fonseca@ee.ufcg.edu.br (N.S.S.M.F.); antonio.leite@ee.ufcg.edu.br (A.F.L.N.); edson@dee.ufcg.edu.br (E.G.C.); 3 Graduate Program in Electrical Engineering, Federal University of Campina Grande (UFCG), Campina Grande 58429900, PB, Brazil; 4 Transmissoras Brasileiras de Energia (TBE), São Paulo 04551000, SP, Brazil; ejunior@tbe.com.br

**Keywords:** lightning, lightning current measurement, discharge counter, overhead power lines monitoring, automation, Rogowski coil

## Abstract

Lightning strikes are a leading cause of outages on overhead transmission lines, significantly compromising power system reliability. Consequently, monitoring lightning activity is critical to mitigate its impact on lines with high outage rates. This study presents an autonomous lightning strike counter system utilizing a split-core Rogowski coil for non-invasive current measurement on transmission towers. The system combines the Rogowski coil with an active integrator circuit to reconstruct the incident current waveform from the coil voltage signal. A microcontroller-based processing unit records strike occurrences and classifies them by amplitude using predefined thresholds. Laboratory tests were carried out to evaluate the performance of the Rogowski coil and integrator circuit, validating the system accuracy in detecting current pulses associated with lightning strikes. Underway field tests will assess the sensor’s reliability during long-term autonomous operation on 345-kV transmission towers. The results demonstrate that the proposed system represents a practical and cost-effective solution for lightning monitoring in remote areas, contributing to enhanced data collection for engineering studies and improved reliability of electrical infrastructure.

## 1. Introduction

Lightning strikes are one of the main causes of outages in overhead power transmission lines [1]. They have a negative impact on power system reliability [2], particularly when tower grounding systems underperform due to design deficiencies or high soil resistivity [3]. Lightning strikes may compromise electrical systems directly through shield wire inadequacies or indirectly through electromagnetic induction or the potential rise of structures, both of which can trigger back flashover [4].

Given the risks lightning strikes pose to power systems, it is essential to characterize their properties and associated direct/indirect effects. However, lightning strikes are complex phenomena that remain challenging to characterize [1,5], as their frequency, duration, polarity, and amplitude exhibit inherent randomness. Pioneering work by Berger, Anderson and Kröninger [6] measured return-stroke current waveforms and established statistical models for these parameters, while [4] proposing additional modeling approaches. These advances are critical for engineering applications, enabling lightning current parameterization. Accurate estimation of lightning strike occurrence rates represents another critical requirement, complementary to current waveform modeling. Although isokeraunic (lightning density) charts offer regional estimates, they often fail to capture localized incidence rates with sufficient precision.

Despite all efforts in this research area, a complete characterization of lightning strikes remains elusive, considering that they are phenomena with inherent stochastic nature associated with climatic conditions, meteorological factors, and local geographical characteristics [1,4,5]. Consequently, continuous research is needed to improve the accuracy of models for transmission line and associated electrical infrastructure design. As a key step, the assessment of lightning strike currents represents a critical research challenge with multiple methodological approaches available.

In the technical literature, various techniques for lightning strike detection have been reported, including the use of current transformers such as Pearson coils [7], electric field sensors [8], inductive sensors [9], optical current transformers [10], electret-based sensors [11], and vibration detectors [12]. In [13], direct measurements of lightning peak currents using magnetic tape were described. That study provided local characteristics of lightning currents and proposed a measurement system consisting of a lightning event counter and magnetic tape.

An alternative is the use of Rogowski coils [8,12,14,15,16,17,18,19], which offer several advantages such as the ability to acquire the current waveform, relatively low cost, linearity, and simple construction. Table 1 provides a comparative overview of typical current sensing technologies, demonstrating how the Rogowski coil achieves a favorable balance between cost, wide current range, fast temporal response, and ease of integration with simple low-power systems, justifying its adoption in the present work.

As highlighted in [20,21], an integrator circuit is required to reconstruct the current waveform from the coil output. However, several challenges must be addressed to ensure the applicability of sensors for lightning strike monitoring. Among these, correcting distortions caused by the frequency response of the coils is particularly critical [20]. Consequently, studies employing Rogowski coils to measure lightning strike waveforms have also proposed noise attenuation methods to enhance measurement accuracy and frequency response [20,22,23,24,25,26,27,28].

In [29], a lightning current measurement instrument incorporating a Rogowski coil and dual signal conditioning circuits was developed. The system was capable of analyzing parameters such as peak current, polarity, rise time, half-wave time, and total charge of the lightning current, as well as distinguishing between induced and direct currents. However, although effective in reducing distortions and characterizing some lightning current parameters, these studies do not address important functionalities such as strike classification by amplitude, event counting over specific time intervals, or data storage capabilities.

Similarly, the authors of [30] developed a low-cost, real-time transient recorder for high-voltage systems. While capable of capturing high-voltage events, the device does not provide features for strike classification, event counting, or mass memory storage.

Furthermore, there is a lack of studies evaluating and demonstrating the effectiveness of split-core Rogowski coils. This type of sensor offers the advantage of enabling non-invasive current measurement on existing tower structures in the field [31]. Another difficulty is the use of sensor systems on towers located in remote areas.

In this sense, this research aims to address current challenges in lightning monitoring by developing and demonstrating a low-cost, stand-alone lightning strike counter system for transmission towers. The system integrates non-invasive current measurement using a split-core Rogowski coil with an active integrator circuit and a digital processing unit. This configuration enables not only the detection of lightning strikes, but also the classification of events by amplitude, counting occurrences over the time it is installed, and storing data in mass memory. Powered by a long-lasting stationary battery, the system ensures autonomous operation and is suitable for deployment in remote locations. The main innovation lies in the combination of these functionalities—non-invasive installation, strike amplitude classification, mass storage data logging, and extended autonomy—in a compact and practical solution, offering clear advantages over existing systems that typically lack this level of integration.

To describe the design and testing of the proposed system, this paper is structured as follows: Section 2 describes the proposed measurement system. Section 3 reports the laboratory tests performed to characterize and evaluate the performance of the Rogowski coils used and the system components. Section 4 reports the field deployment of the prototypes. The conclusions are presented in Section 5.

## 2. Proposed Lightning Strike Counter System

### 2.1. Flowchart of the Proposed System

**Figure 1:**
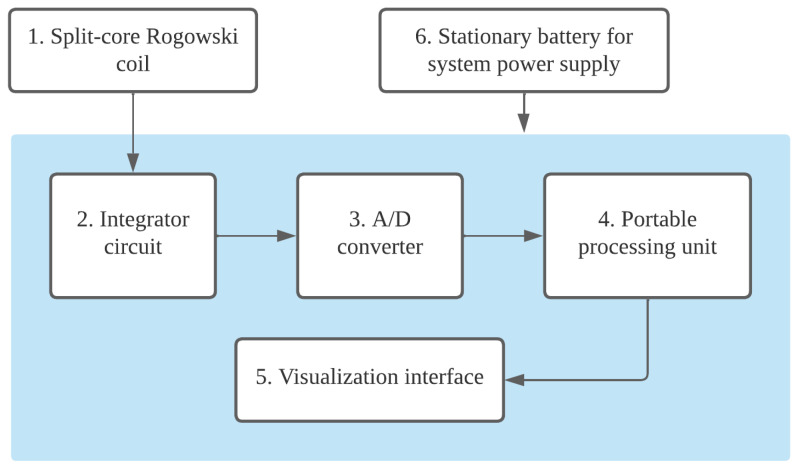
Block diagram of the autonomous system developed for recording and counting lightning strikes.

Figure 1 presents the block diagram of the autonomous system developed for recording and counting lightning strikes. The system is designed to monitor lightning strikes on a power transmission tower and is organized into six modules, each responsible for the functions detailed in the figure.

The following subsections describe the system components and outline the design steps and assumptions that guided their specification.

### 2.2. Transducer Selection, Characterization, and Performance Evaluation

By measuring the current through one of the tower legs, it is possible to detect a lightning strike. For this purpose, a Rogowski coil current transducer was employed, as it is well-suited for measuring high-amplitude currents.

Since these transducers do not contain a ferromagnetic core, they are immune to magnetic saturation, allowing for highly linear current measurements across wide frequency and amplitude ranges [32,33]. In addition, they can be built with open-core designs, facilitating field installation without requiring modifications to the monitored structure, thereby enabling non-invasive sensing.

The Rogowski coil encircles a conductor carrying a time-varying current i(t). According to Ampere’s law [5], the current i(t) generates a magnetic field in the space adjacent to the conductor, such that the line integral of the magnetic field $\left(\int \vec{H}\cdot d\vec{l}\right)$ is equivalent to i. Simultaneously, according to Faraday’s law [5], a voltage proportional to the rate of change of the enclosed magnetic flux (and, thus, proportional to the rate of change of the current i) is induced in the coil. As a result, the coil converts an incident high-amplitude current signal into a low-amplitude voltage signal at its output. The relationship between output voltage and input current in the coil is expressed by Equation (1):

(1) $$v_{\mathrm{out}}=-M\frac{di(t)}{dt},$$

where $v_{\mathrm{out}}$ is the voltage across the coil output terminals, i(t) is the monitored current, and *M* is the mutual inductance between the coil and the conductor it encircles. The value of *M* can be provided by the manufacturer or calculated based on the coil parameters, primarily its cross-sectional area, number of turns, and length.

Following preliminary frequency response tests, a commercial split-core Rogowski coil was selected for the prototype implementation. Its technical specifications are presented in Table 2.

It is worth noting that, even if the transducer manufacturer specifies the capability to measure currents up to 300 kA, the system was not tested across that entire range due to the limited maximum current amplitude achievable by the impulse current generator used. As a result, it was not possible to verify the linearity of the transducer–integrator system throughout the full range specified by the manufacturer. However, this does not compromise the intended functionality of the system, which is not designed to determine the exact amplitude of the strike current, but rather to classify it into predefined value ranges, as described in Section 2.4.

As described in Table 2, the coil exhibits a frequency bandwidth of 420 kHz, defined as the frequency at which the attenuation reaches −3 dB. This bandwidth is considered adequate for lightning current measurements, as it encompasses nearly the entire frequency content associated with standardized waveforms for both direct and indirect lightning strikes [34].

**Figure 2:**
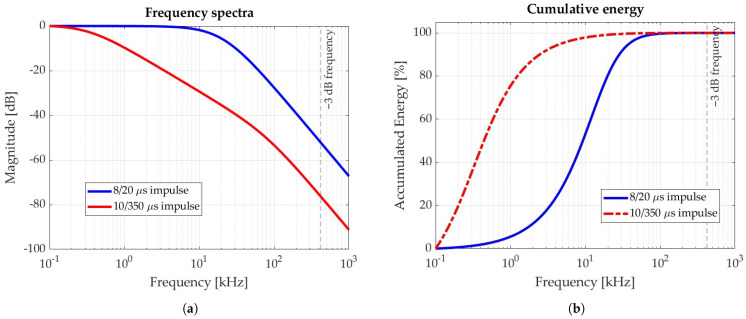
Frequency domain analysis of standardized 8/20 μs and 10/350 μs impulses: (**a**) Frequency spectra. (**b**) Cumulative energy content.

To illustrate this, Figure 2 presents a frequency-domain analysis of the 8/20 μs and 10/350 μs impulses—waveforms commonly used to represent indirect and direct lightning strikes, respectively. The analysis was performed using the double exponential model [34] to represent lightning strike current i(t):

(2) $$i\left(t\right)=I_{0}\left(e^{-at}-e^{-bt}\right),$$

with the corresponding parameter values provided in Table 3. Figure 2a compares the spectral content of the standardized impulses with the coil −3 dB cutoff frequency, showing that the attenuation at 420 kHz reaches −52 dB (corresponding to less than 0.3% of the DC component) for the 8/20 μs waveform, and −76 dB (less than 0.02%) for the 10/350 μs waveform.

Figure 2b shows the cumulative energy distribution for both waveforms. In each case, more than 99.99% of the total energy is concentrated below the coil −3 dB cutoff frequency, confirming the suitability of the coil for accurately capturing the relevant spectral components of lightning currents.

### 2.3. Active Integrator Circuit

Since the output voltage signal of the Rogowski coil is proportional to the derivative of the monitored current, an integrator circuit is required to recover the original current waveform. Active integrators are typically employed, as they provide signal amplification and operate over a broader frequency range (0–4 MHz). The integrator transfer function must compensate for the Rogowski coil frequency response, ensuring the combined system exhibits a flat magnitude response and near-zero phase shift within the lightning current spectral range [35].

**Figure 3:**
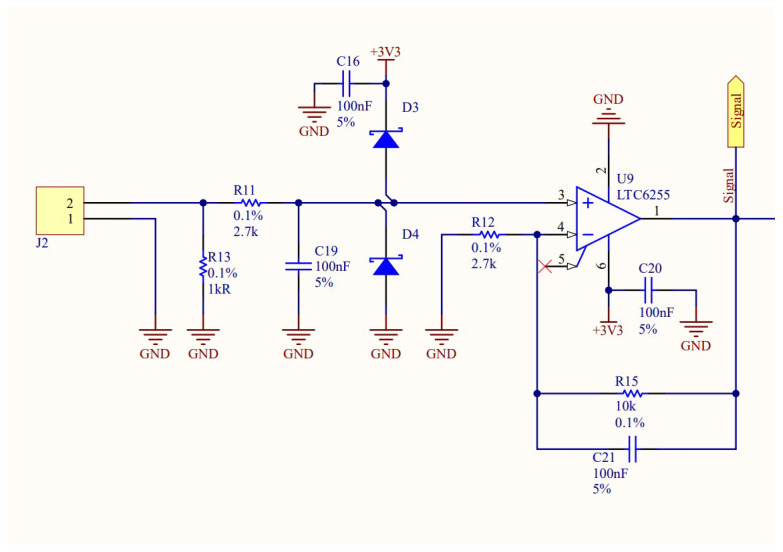
Diagram of the active integrator circuit implemented.

In this research, an active integrator configuration was used [36]. The circuit consists of an operational amplifier with a capacitor and a resistor connected in parallel in the feedback loop, forming a negative feedback network. A resistor is connected between the inverting input and ground. The voltage signal from the Rogowski coil is applied to the non-inverting input of the operational amplifier, and the output provides the integrated voltage, which is directly proportional to the measured current. A detailed schematic of the implemented integrator circuit is presented in Figure 3. In addition to the integrator stage, the circuit includes additional capacitors for voltage regulation and voltage limiters to ensure stable and reliable operation.

### 2.4. Definition of Threshold Values and Impulse Detector Module

A threshold-based procedure was employed to identify the occurrence of a lightning strike. To this end, an impulse detector was developed, consisting of an analog circuit connected to an analog-to-digital converter (ADC). This detector continuously monitors the voltage signal from the inductive current sensor and compares it against predefined threshold levels. By appropriately defining these thresholds, it is possible not only to detect lightning strikes but also to classify them according to specific amplitude ranges.

The impulse detector circuit was built with three thresholds, as detailed in Table 4. According to the statistical model presented in [37], 97% of lightning strikes have a peak value greater than 600 A, which was adopted as the first threshold for defining the occurrence of a lightning strike. The second and third thresholds were defined as 5 kA and 10 kA, respectively, enabling the study of the intensity of lightning strikes that typically affect the monitored structure.

**Figure 4:**
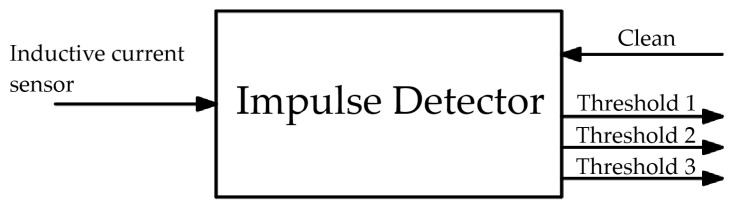
Schematic diagram of the impulse detector module.

In Figure 4, a schematic diagram of the impulse detector module is shown. The circuit receives the voltage signal from the current inductive sensor at its input. The impulse detector is connected to a portable processing unit via four digital ports. When a lightning strike is detected, the impulse detector activates one of the three threshold ports to indicate its occurrence and classify its amplitude range. The portable processing unit logs the event and sends a signal to the impulse detector through the reset port to return it to the alert mode.

**Figure 5:**
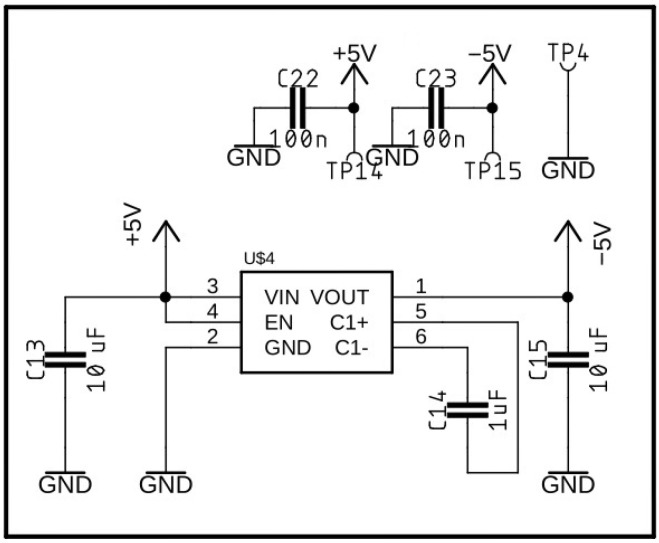
Circuit diagram of the negative voltage source.

**Figure 6:**
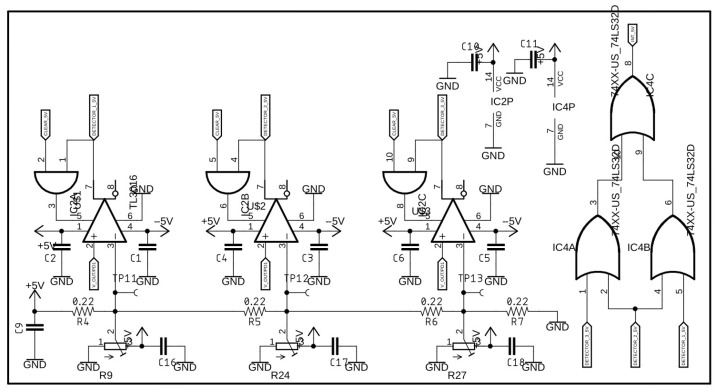
Circuit diagram of the impulse detector module.

**Figure 7:**
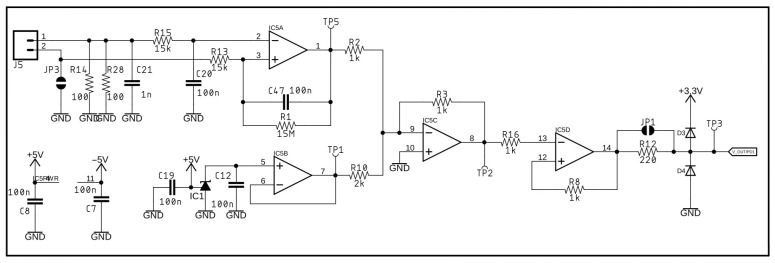
Diagram of the analog signal conditioning circuit.

Figure 5, Figure 6 and Figure 7 present circuit diagrams of the main components of the impulse detector module, namely: the negative voltage source (Figure 5), the impulse detector (Figure 6), and the analog signal conditioning circuit (Figure 7).

### 2.5. Portable Processing Unit

The portable processing unit (block 4 in Figure 1) is responsible for storing the records obtained from the impulse detector and controlling a visualization interface, which enables users to observe the total number of strikes and the peak value of each individual strike. In the current prototype version, lightning strike data—including occurrence, date, time, and amplitude range—is stored locally on a microSD card. This allows field teams to retrieve data during scheduled inspections. Although wireless communication is not implemented in this version, the system’s microcontroller architecture enables future integration with communication modules such as LoRa, NB-IoT, or GSM. However, for remote locations where signal coverage is limited and maintenance logistics are complex, local data storage remains a practical and robust solution.

The system has a low power consumption of about 2.3 mW in standby mode, determined from bench tests. In standby mode, only the current inductive sensor and the impulse detector in alert mode remain continuously active. The portable processing unit is activated only when a lightning strike is detected, returning to hibernation mode shortly after logging the event.

The field-installed system version is powered by a 12 V, 7 Ah stationary battery, which ensures system autonomy for approximately 20 months. This value was estimated based on the standby mode power consumption and assuming a monthly self-discharge rate of 2% [38], which results in a self-discharge power of about 2.3 mW. In addition, a maximum discharge depth of 80% was considered to preserve the battery long-term service life. This estimated autonomy comfortably covers the scheduled inspection interval of six months. To ensure continued system operation after the maximum autonomy period, the battery should be replaced.

In a possible continuation of this research, improvements in energy supply are planned by exploring potential power optimization strategies. These include implementing a deep sleep mode [9,39], using higher-capacity batteries, or integrating alternative energy sources to extend system autonomy. One possible strategy is to incorporate a solar panel in conjunction with the stationary battery, allowing continuous system operation over extended periods.

**Figure 8:**
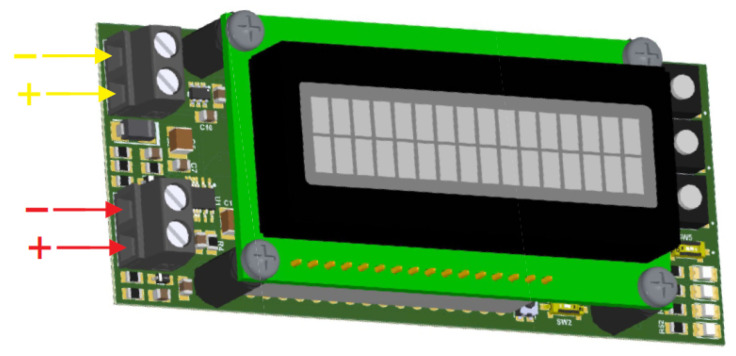
Illustration of the lightning counter system prototype (red arrows: Rogowski coil connection; yellow arrows: battery connection).

Additionally, the system has a 3 V lithium battery, responsible for keeping the microcontroller clock running even in the event of an interruption in the power supply from the main battery. A prototype of a printed circuit board (PCB) was designed and manufactured to accommodate the active integrator, the A/D converter, the portable processing unit and the visualization interface, resulting in a prototype as shown in Figure 8. Externally to the PCB, the Rogowski coil is connected to the red-marked sockets, while the stationary battery is connected to the yellow-marked sockets.

### 2.6. Prototype Connection Mode to the Transmission Tower Structure

**Figure 9:**
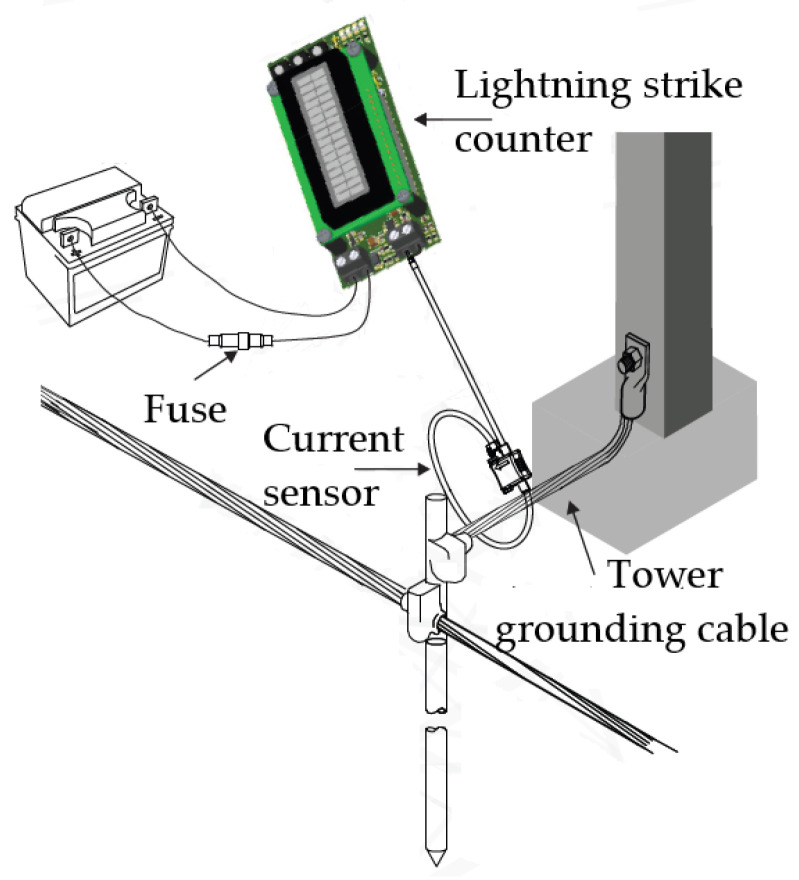
Schematic diagram of the circuit connection to the high-voltage tower.

Figure 9 presents a schematic diagram illustrating how the circuit should be connected to the transmission tower for lightning strike monitoring. The Rogowski coil encircles the grounding cable of one of the tower legs, and its output terminals are connected to the PCB. The stationary battery is also connected to the PCB through a fuse, which protects the circuit against current surges that may occur during battery connection.

## 3. Laboratory Tests Performed to Evaluate System Performance

To evaluate the performance of the lightning strike counter system in detecting high-amplitude current surges, tests were carried out using the impulse current generator (ICG) at the High Voltage Laboratory (LAT) of the Federal University of Campina Grande (UFCG). This section presents the test procedures and discusses the results obtained.

### 3.1. Material Used for Testing

**Figure 10:**
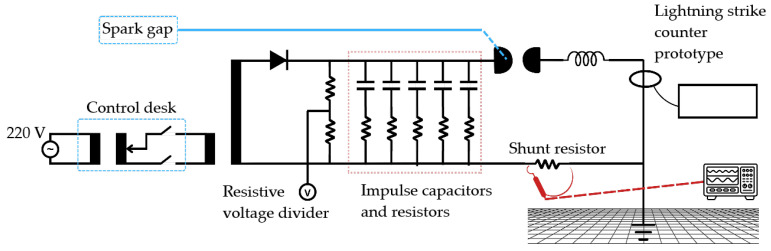
Schematic diagram of the ICG at LAT-UFCG.

The main components of the ICG at LAT-UFCG are shown in the schematic diagram of Figure 10: a high-voltage transformer (220 V/100 kV), a high-voltage rectifier diode, and a configurable bank of high-voltage capacitors.

**Figure 11:**
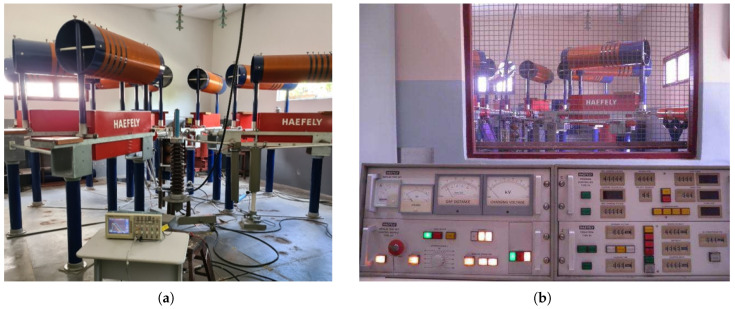
Photographs of the ICG setup and control desk shown: (**a**) ICG room interior. (**b**) ICG view and control desk.

The ICG was used to produce current impulses representative of lightning strike currents for testing. In addition to the capacitors, the generator includes series resistors and an inductor to shape the waveform, with the current measured indirectly by a shunt resistor, as shown in Figure 10. In the tests, five 2.0 μF capacitors were used, each connected in series with a 5.0 Ω resistor, and the inductance value was 5.7 μH. Illustrative photographs of the setup and control desk are shown in Figure 11.

### 3.2. Tests Performed

The tests consisted of applying current impulses to the ICG grounding grid. The resulting current was measured indirectly using a 5.488 mΩ Park-type shunt resistor. The voltage across the resistor was monitored using a probe connected to an oscilloscope. During each impulse, the oscilloscope signal was observed to determine the voltage amplitude across the shunt, as well as the front and tail times of the waveform. The current amplitude was obtained by dividing the voltage amplitude across the resistor by its resistance.

Two types of tests were performed:

- **Test of the Rogowski coil output**: in this step, the current waveforms obtained from the ICG control desk were compared with those computed by integrating the coil output signal. The objective was to verify the linearity of the measurement system under development;
- **Evaluation of the complete counter prototype**: in this step, the ability of the counter prototype to reliably detect and classify lightning strikes according to the predefined amplitude ranges in Table 4 was evaluated. For each of the four ranges, a series of 10 current impulses was applied. The aim was to verify whether the counter correctly identified and recorded the number of impulses falling into each amplitude range. The voltage across the shunt resistor, measured from the oscilloscope, was used as a reference to determine the true current amplitude in each test. This procedure allowed a direct validation of the classification function of the sensor.

### 3.3. Results Obtained from Laboratory Tests

**Figure 12:**
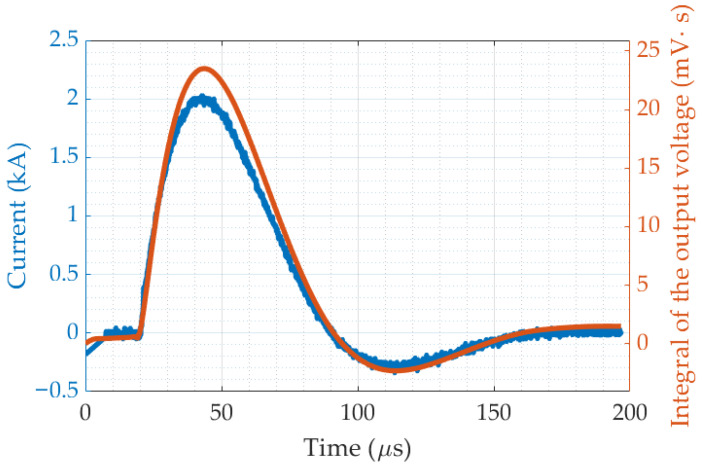
Shunt-resistor current (blue) and integral of Rogowski coil output voltage (orange).

**Figure 13:**
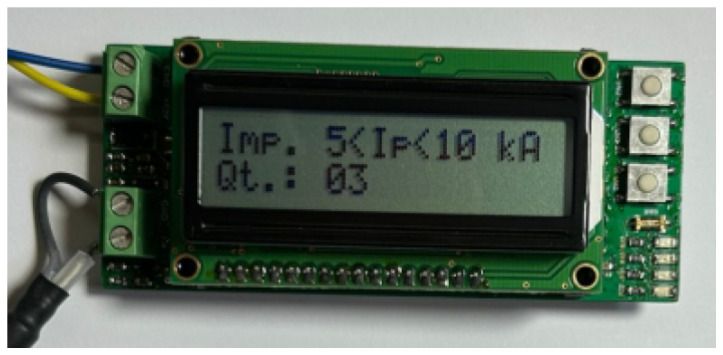
Example photograph of the interface showing three current impulses in the 5 kA to 10 kA range.

- **Test of the Rogowski coil output**: The current waveform measured using the IGC shunt resistor was compared with the current waveform obtained from the integration of the Rogowski coil output signal. The results demonstrated waveform alignment, differing only by a scaling factor related to the coil transformation ratio. To analytically assess the linearity of the coil-integrator system, the Pearson’s correlation coefficient [40] between the two signals was calculated, yielding *r* = 0.998. This high correlation confirms a strong linear relationship between the reference current waveform and the current derived from the Rogowski coil integration process. As an example, a comparison of the two waveforms is presented in Figure 12.
- **Evaluation of the complete counter prototype**: after connecting the lightning counter prototype to the ICG grounding cable and performing multiple series of current impulse applications across the defined amplitude ranges, it was verified that the counter correctly recorded all 10 impulses in each of the four series, with no misclassification. The current amplitude measured via the shunt resistor consistently matched the amplitude range recorded using the counter, demonstrating the system reliability in identifying and registering lightning events according to the proposed thresholds. Figure 13 presents an example of three current impulses recorded in the range of 5 kA to 10 kA.

## 4. Field Deployment of the Prototypes

In the final stage of the project, nine prototypes developed and tested in the laboratory were installed on a pilot transmission line located in the state of Minas Gerais, Brazil. The devices were mounted on towers classified as non-compliant—that is, towers assessed as being more exposed to lightning strikes or presenting high grounding resistance values. The prototypes were enclosed in cylindrical PVC inspection boxes for protection.

**Figure 14:**
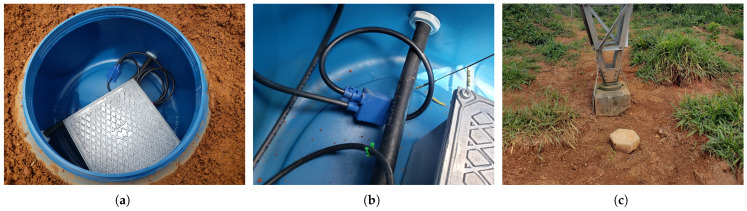
Photographs showing the installation process of one of the lightning strike counter systems on a transmission line tower: (**a**) Prototype inside the box. (**b**) Detail of the Rogoswki coil encircling the grounding cable. (**c**) Concrete block protecting the system.

Figure 14 shows photographs of the installation process for one of the systems. Figure 14a depicts the lighting counter system housed inside its inspection box. The polymeric enclosure contains the circuit and the stationary battery, with a Rogowski coil cable as output. Figure 14b provides a closer view of how the coil is connected to the grounding cable. After installation, the inspection box was sealed with a hermetic lid to protect the system from water, humidity, and animals. Finally, a concrete block was placed over the lid to provide additional protection to the system, as shown in Figure 14c. This installation procedure was repeated for all nine towers equipped with lightning counters.

While this study focused primarily on the design, development, and laboratory validation of an autonomous lightning strike counting system for transmission towers, it is worth noting that the current prototype already meets several normative requirements specified in IEC 62561-6 [41], including:

- **Environmental resilience**: Operational temperature range from −40 °C to +85 °C; IP67-rated enclosure providing dust and water protection;
- **Threshold detection**: Configurable current threshold, set to 0.6 kA in the current implementation;
- **Display readability**: Strike count displayed using 16 mm-high characters, ensuring clear visibility;
- **Safe installation**: Stress-free mounting on the conductor enabled by the split-core Rogowski coil design.

Future work will systematically validate the system against all criteria outlined in IEC 62561-6, with particular focus on long-term performance and reliability. The field deployment enables in situ testing under real environmental conditions—including temperature fluctuations, humidity, vibration, and other external factors—which will support a comprehensive assessment of the system robustness and stability over time.

## 5. Conclusions

This article described and discussed the design, development, and laboratory testing of an autonomous system for recording and counting lightning strikes on transmission lines. The system consists of a Rogowski coil combined with an integrator circuit, and is powered by a stationary battery. It can count the number of lightning strikes on a specific tower, register data and time of occurrence, and classify them according to their amplitude.

The proposed system provides critical monitoring data for transmission lines prone to frequent outages, enabling identification of the most vulnerable structures and estimation of lightning strike current amplitudes on impacted towers. Laboratory tests demonstrated the effectiveness of the proposed system, which was optimized to meet operational requirements for electromagnetic compatibility and low power consumption, thereby extending its operational autonomy.

In the final phase of the project, nine lightning strike counters were successfully installed on 345-kV transmission towers in Minas Gerais, Brazil. Sensors performance will be evaluated under field operating conditions. This implemented solution represents a significant advancement in power transmission automation, providing improvements in power system reliability through enhanced lightning monitoring capabilities.

## Figures and Tables

**Table 1:** Comparison of current sensing technologies for lightning monitoring applications.

Criterion	Split-Core Rogowski Coil	Pearson Coil	Optical Sensor	Shunt/Hall-Effect
Installation	Non-invasive	Requires circuit interruption	Non-invasive	Invasive/Direct contact
Power Supply Needed	No	No	Yes	No/Yes
Cost (USD)	Low (<100)	High (500–1000)	Very High (1000–5000)	Low–Medium (50–300)
Field Suitability	Excellent	Moderate	High (complex)	Poor/Limited
Current range	Very wide	Moderate	Wide	Moderate

**Table 2:** Electrical data of the split-core Rogowski coil.

Parameter	Value	Unit
Measurement range	Up to 300	kA
Mutual inductance	72.0	nH
Transformation ratio at 50 Hz	22.5	mV/kA
Frequency bandwidth (−3 dB)	420	kHz
Ratio error (all positions)	±0.75	%

**Table 3:** Double exponential model parameters for standardized lightning impulses.

Impulse	Parameters	
	a ** (s^−1^)**	b ** (s^−1^)**
8/20 μs	$1.203\times 10^{5}$	$1.430\times 10^{5}$
10/350 μs	$2.073\times 10^{3}$	$4.991\times 10^{5}$

**Table 4:** Thresholds adopted for lightning strike current classification.

Region	Description
1	$I_{p}$ < 0.6 kA (Discharge is not registered)
2	0.6 kA $\leq I_{p}$ < 5 kA
3	5 kA $\leq I_{p}<$ 10 kA
4	$I_{p}\geq$ 10 kA

## Data Availability

The raw data supporting the conclusions of this article will be made available by the authors on request.

## Abbreviations

The following abbreviations are used in this manuscript:

ADC	Analog-to-Digital Converter
ICG	Impulse Current Generator
IEC	International Electrotechnical Commission
LAT	Laboratório de Alta Tensão (High-Voltage Laboratory)
PCB	Printed Circuit Board
UFCG	Federal University of Campina Grande
